# Adipokines as Biomarkers in Health and Disease

**DOI:** 10.1155/2018/5696815

**Published:** 2018-10-04

**Authors:** Julie Bienertova-Vasku, Manlio Vinciguerra, Marek Buzga, Francesc Villaroya

**Affiliations:** ^1^Research Centre for Toxic Compounds in the Environment (RECETOX), Faculty of Science, Masaryk University, Brno, Czech Republic; ^2^Department of Pathological Physiology, Faculty of Medicine, Masaryk University, Brno, Czech Republic; ^3^International Clinical Research Center, St. Anne's University Hospital, 656 91 Brno, Czech Republic; ^4^Institute for Liver and Digestive Health, Division of Medicine, University College London (UCL), London, UK; ^5^Research Obesity Centre, Faculty of Medicine, University of Ostrava, Ostrava, Czech Republic; ^6^Departament de Bioquímica i Biomedicina Molecular and Institut de Biomedicina (IBUB), Universitat de Barcelona, Barcelona, Spain; ^7^CIBER Fisiopatología de la Obesidad y Nutrición, Barcelona, Spain

Adipokines are important circulating factors mediating intertissue crosstalk throughout the body and thus playing a key role in maintaining endocrine homeostasis. So far, multiple associations of adipokines with widespread noncommunicable diseases, including cardiometabolic diseases, cancer, neurodegenerative diseases, and fertility problems, have been identified. However, the precise mechanisms underlying empirically observed associations are still rather poorly understood, and more research must be carried out in order to make sense of and deepen the already published data.

A better understanding of associations of adipokine expression and common diseases seems warranted, and the journal set out to publish this special issue. The preparation of this special issue resulted in a series of 5 articles, submitted by researchers from Kuwait, Czech Republic, Germany, and China. All the published studies were approved by the local Ethical Committees, and the statements are included in the Method section of the respective papers.

First, T. Pavlova et al. (Czech Republic), in their work titled “Irisin Maternal Plasma and Cord Blood Levels in Mothers with Spontaneous Preterm and Term Delivery,” focused on the possible association of irisin levels—a protective exercise-induced myokine—with preterm birth and concluded that irisin levels in cord blood were significantly different between preterm and term pregnancies. To the best of our knowledge, this is the first study ever to report the association of irisin cord blood levels with preterm birth.

Second, A. Koch et al. (Germany) in their work titled “Visfatin Serum Levels Predict Mortality in Critically Ill Patients” focused on the potential of visfatin to predict mortality of critically ill patients in the intensive care unit (ICU). Visfatin is an adipokine also called nicotinamide phosphoribosyltransferase (NAMPT), which displays insulin-mimicking effects. The authors report that the visfatin serum concentrations were strongly associated with disease severity and organ failure, irrespectively of the possible presence of type 2 diabetes mellitus (T2DM). Moreover, the authors observed that visfatin levels correlated with biomarkers of renal failure, liver dysfunction, and other adipokines (e.g., resistin, leptin, and adiponectin) in their cohort. Last but not least, high visfatin levels at ICU admission were predictive of an increased mortality, both at the ICU and during a long-term follow-up of approximately two years. Such findings emphasize strongly the need for a replication study of the potential of visfatin for the prediction of organ failure and/or overall survival in ICU patients.

Lastly, three papers focused on the possible associations of specific adipokines with insulin sensitivity or T2DM with or without concomitant metabolic syndrome: (i) F. Wang et al. from China (manuscript titled “Circulating PGRN Levels Are Increased but Not Associated with Insulin Sensitivity or β-Cell Function in Chinese Obese Children”) investigated the association of progranulin with insulin sensitivity or *β*-cell function in a Chinese paediatric cohort, (ii) N. A. Abdella and O. A. Mojiminiyi from Kuwait (manuscript titled “Clinical Applications of Adiponectin Measurements in Type 2 Diabetes Mellitus: Screening, Diagnosis, and Marker of Diabetes Control”) focused on adiponectin as a potential diagnostic marker for T2DM or insulin resistance, and (iii) P. Yan et al. from China (manuscript titled “Plasma Neuregulin 4 Levels Are Associated with Metabolic Syndrome in Patients Newly Diagnosed with Type 2 Diabetes Mellitus”) investigated the link between neuregulin 4 and metabolic syndrome in newly diagnosed T2DM patients. Each of these works provides unique insights into the field, suggesting, respectively, that (i) progranulin is associated with higher BMI and multiple parameters of obesity in obese children, (ii) adiponectin may be useful as possible diagnostic/therapeutic markers in T2DM, and (iii) neuregulin 4 is associated with parameters of metabolic syndrome in newly diagnosed T2DM patients.

Taken together, the presented papers further emphasize the complexity of the adipokine network that likely serves as master relays for integrating the influences of environmental factors with the genetic and epigenetic makeup of every individual. All the included papers further highlight the importance of larger prospective studies as well as validation studies on different populations of patients and different ethnicities. To conclude, the presented special issue provides a collection of novel findings in the field, contributing possibly to better decision-making in the clinical practice. It should be kept in mind, however, that adipokines are part of large neuroendocrine-immune-metabolic networks, and they exert their effects through complex endocrine, paracrine, autocrine, or juxtacrine crosstalk mechanisms, which should be further investigated with system medicine approaches.

